# Case Report: A rare culprit of severe pulmonary infection in children: prevotella

**DOI:** 10.3389/fped.2026.1782202

**Published:** 2026-03-19

**Authors:** Yijun Cheng, Li Peng, Dan Liu, Lili Zhong, Yao Liu, Tuhong Yang

**Affiliations:** 1 The First Affiliated Hospital of Hunan Normal University Hunan Provincial People’s Hospital, Changsha, China; 2 Children's Medical Center, Hunan Provincial People’s Hospital and The First-affiliated hospital of Hunan Normal University, Changsha, China

**Keywords:** Prevotella, pulmonary infection, severe, metagenomic next-generation sequencing, children, treatment, pulmonary rehabilitation

## Abstract

**Background:**

To characterize the clinical features, diagnostic pitfalls, and treatment of severe pediatric pulmonary infection caused by Prevotella species.

**Methods:**

We retrospectively reviewed clinical data, the diagnostic workflow, antimicrobial regimens, and outcomes of two children with severe Prevotella pulmonary infection.

**Results:**

Case 1 was an 11-year-old boy with necrotizing pneumonia, and Case 2 was a 13-year-old boy with retained foreign-body aspiration. Both patients responded poorly to initial cephalosporin-based therapy. Metagenomic next-generation sequencing (mNGS) of bronchoalveolar lavage (BAL) fluid identified Prevotella nanceiensis (sequence count 299,022; relative abundance 92.24%) and Prevotella oralis (210,449; 67.98%) within 24 h, whereas anaerobic culture (Case 1) became positive after 4 days. Based on mNGS results antibiotics were adjusted to metronidazole plus a carbapenem ( meropenem for Case 1; imipenem-cilastatin for Case 2), and both children received adjunctive pulmonary rehabilitation before discharge. They subsequently recovered and were discharged.

**Conclusion:**

Severe Prevotella pulmonary infection in children has non-specific manifestations and may respond poorly to conventional beta-lactam therapy, leading to delayed diagnosis. mNGS enables rapid pathogen identification and supports targeted anti-anaerobic treatment. For severe or complicated cases refractory to empirical therapy, metronidazole combined with a carbapenem may be an effective option.

## 1 Introduction

Prevotella spp., previously classified within Bacteroides, are obligate anaerobic Gram-negative coccobacilli that form part of the normal flora of the oral cavity and the upper respiratory and genitourinary tracts (1). Under conditions such as impaired host defenses or disrupted mucosal barriers, Prevotella can cause invasive infections, including severe pulmonary infection. In children, clinical manifestations are often non-specific and early empirical beta-lactam therapy may be ineffective, increasing the risk of delayed diagnosis. Therefore, when severe pneumonia fails to respond to conventional antibiotics, Prevotella infection should be considered and prompt pathogen-directed testing should be performed to guide antimicrobial adjustment. Here, we report two pediatric cases: (1) necrotizing pneumonia associated with Prevotella nanceiensis and (2) severe pneumonia due to Prevotella oralis in the setting of a retained airway foreign body. These cases highlight the value of early etiologic identification and individualized management.

## 2 Case report

### 2.1 General information

**Figure 1:**
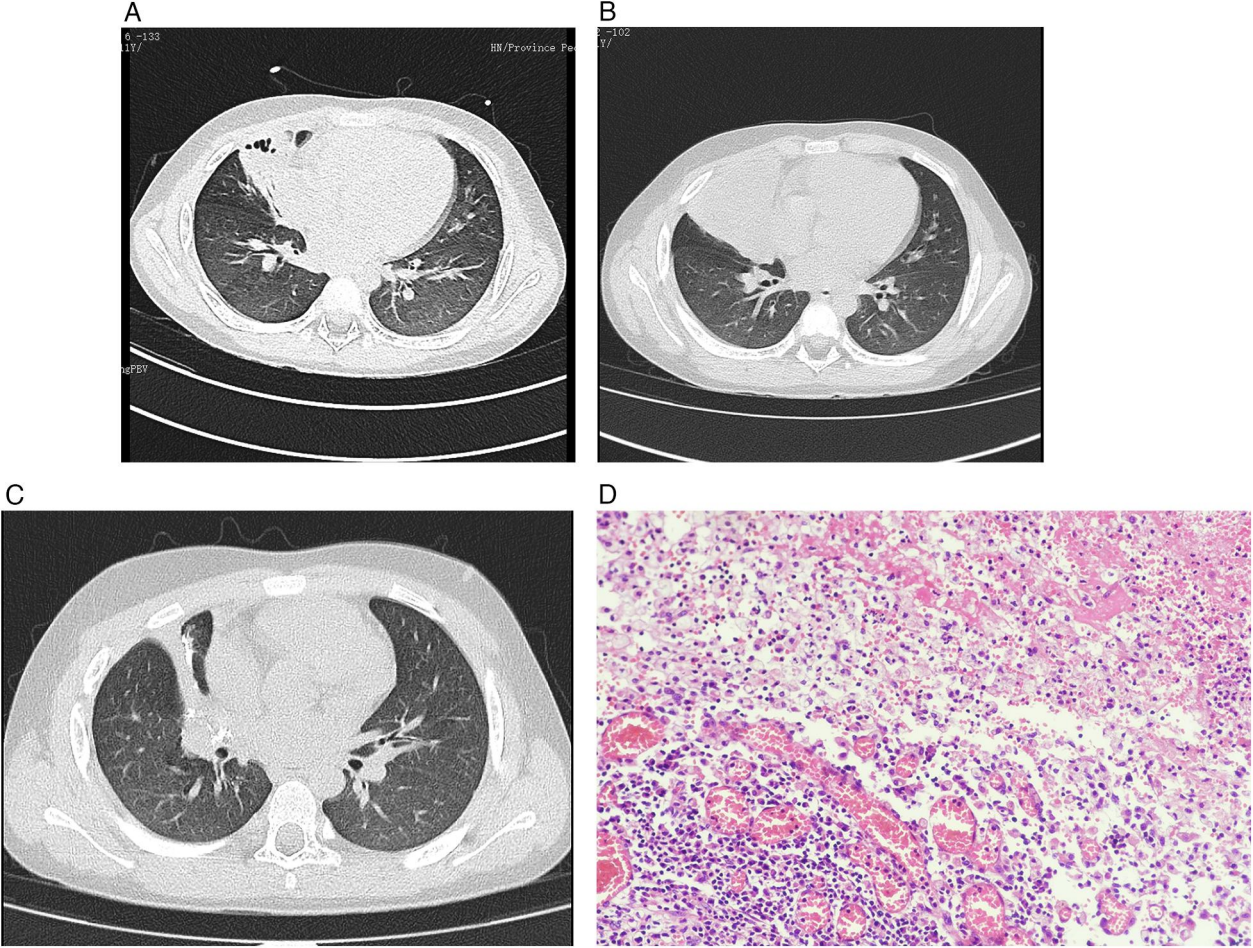
**(A)** Initial chest CT at an external hospital showing inflammation with partial consolidation of the right middle lobe; **(B)** chest CT upon admission showing enlarged lesions in the right middle lobe, local bronchial obstruction, and consolidation (severe imaging manifestations); **(C)** reexamination of chest CT after discharge showing a small amount of chronic inflammation in the right lung and thickening of the pleura in the surgical area; **(D)** HE staining of lung tissue: inflammatory lesions in the right chest and right lung, with hemorrhage, necrosis, abscess formation in the lesion area, and infiltration of a large number of acute and chronic inflammatory cells and histiocytes.

Case 1: An 11-year-old boy was admitted to the hospital due to “recurrent cough and fever for more than 1 month accompanied by chest pain for 9 days”. The child developed fever more than 1 month ago, with body temperature fluctuating between 38–39℃, accompanied by severe cough. An external hospital detected MP-Ab 1:1280, C-reactive protein (CRP) 30.77 mg/L (normal reference value 0–10 mg/L), and white blood cell (WBC) count 11.2 × 10^9^ /L (normal reference value 4∼10 × 10^9^ /L). Chest CT showed right middle lobe consolidation and a small amount of right pleural effusion (Figure 1A). The child was treated with amoxicillin, azithromycin for anti-infection, and methylprednisolone. However, the child's condition was complicated by recurrent fever and aggravated cough. Nine days ago, he developed right chest pain, and the cough worsened with blood-tinged sputum, so he was transferred to our hospital for further treatment. There were no special personal or family histories. Physical examination: Temperature 37.7℃, pulse 102 beats/min, respiratory rate 28 breaths/min, blood pressure 119/76 mmHg. Breath sounds were decreased in the right middle and lower lungs. Auxiliary examinations: Blood routine showed WBC count 18.59 × 10^9^ /L (normal reference value 4–10 × 10^9^ /L), CRP 80.29 mg/L (normal reference value 0–10 mg/L). Repeated chest CT showed an increased range of lesions in the right middle lobe, local bronchial obstruction of the right middle lobe, and consolidation of the middle lobe lung tissue (Figure 1B). Flexible bronchoscopy revealed poor ventilation in the right middle lung with exudation of purulent secretions, and bronchoalveolar lavage fluid was collected for examination. Etiological examination at the external hospital before admission indicated that Mycoplasma pneumoniae was a drug-resistant strain, so levofloxacin was administered for anti-infection upon admission. However, after admission, the child still had recurrent fever, chest pain, and massive hemoptysis, and necrotizing pneumonia was considered to have developed. Video-assisted thoracoscopic right middle lobectomy + pleural adhesion lysis was performed under general anesthesia. Pathological results showed severe acute inflammation of the lung tissue (Figure 1D). At this time, metagenomic next-generation sequencing (mNGS) of bronchoalveolar lavage fluid reported Prevotella nanceiensis (sequence count 299,022) with a relative abundance of 92.24%. For strain identification, bronchoalveolar lavage fluid was used as the specimen. After thorough vortexing, DNA extraction and sequencing library construction were performed per the kit's standard protocol. Sequencing, raw data conversion and sample demultiplexing were conducted on the ONT platform with matching software. Data filtering removed short and low-quality reads, and specific alignment software eliminated host sequences. Finally, microbial alignment, verification and species abundance calculation were performed to complete strain identification. Four days later, anaerobic culture of bronchoalveolar lavage fluid also confirmed Prevotella nanceiensis. Combined anti-infection treatment with metronidazole and meropenem was administered. Half a month later, repeated infection indicators were normal, and repeated chest CT (Figure 1C) showed significant absorption and improvement of right lung inflammation compared with before.

**Figure 2:**
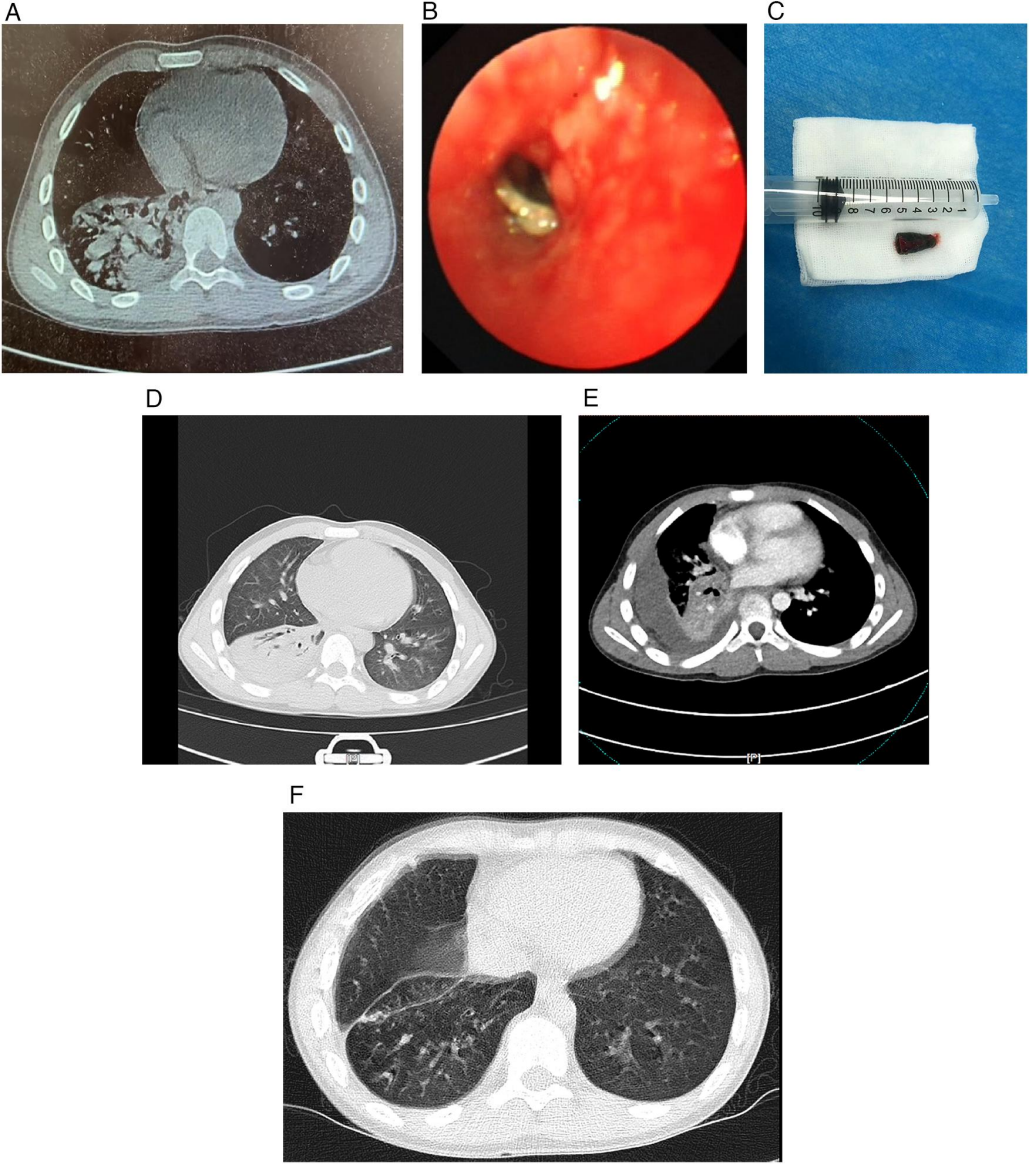
**(A)** initial chest CT at an external hospital showing right lower lobe pneumonia; **(B,C)** images of the pen cap foreign body under bronchoscopy (predisposing factor for severe infection); **(D,E)** chest CT showing patchy high-density shadows in the right lower lobe and right pleural effusion (severe complex pneumonia); **(F)** follow-up chest CT 1 month after discharge showing significant absorption of right lower lobe inflammation and good lung recruitment.

Case 2: A 13-year-old boy was admitted to our hospital due to “cough for 1 week and fever for 2 days”. The child developed cough without obvious inducement 1 week ago, which was more severe at night, accompanied by yellow mucoid sputum and occasional right lower chest pain. Two days ago, he developed fever (body temperature 39℃). Chest CT at an external hospital (Figure 2A) showed “right lower lobe pneumonia”. Bronchoscopy indicated a foreign body in the basilar trunk bronchus of the right lower lobe, and the child was transferred to our hospital for further treatment after an unsuccessful attempt at removal. Past history: Upon repeated inquiry, there was a suspected history of “pen cap” aspiration 2 years ago. He had recurrent respiratory infections in the past 2 years, but anti-inflammatory treatment was effective. There were no special personal or family histories. Physical examination: Temperature 36.6℃, heart rate 94 beats/min, respiratory rate 22 breaths/min, blood pressure 105/58 mmHg. The child was in good spirits, with symmetrical bilateral respiratory movement. Dull percussion sound and decreased breath sounds were noted in the right lower lung, and a few moist rales could be heard. Auxiliary examinations: CRP 13.02 mg/L (normal reference value 0–10 mg/L), WBC count 10.89 × 10^9^ /L (normal reference value 4–10 × 10^9^ /L). Cefotaxime was administered for anti-infection on the day of admission. Under general anesthesia, flexible bronchoscopy was performed to remove the endobronchial foreign body (pen cap) (Figures 2B,C), along with bronchoalveolar lavage and granulation tissue clearance. On the next day, the child still had chest pain. Repeated chest CT showed patchy high-density shadows in the right lower lobe and right pleural effusion (Figures 2D,E). At this time, mNGS of bronchoalveolar lavage fluid detected Prevotella oralis with a sequence count of 210,449 and a relative abundance of 67.98%. After the mNGS result was reported, the anti-infection treatment was changed to metronidazole and imipenem-cilastatin sodium, and thoracentesis was performed to aspirate pleural fluid. mNGS of pleural fluid still indicated Prevotella oralis with a sequence count of 17,571 and a relative abundance of 98.80%. After 12 days of anti-infection treatment, the symptoms improved, repeated infection indicators were normal, and the child was discharged in good condition. One month after discharge, outpatient reexamination of chest CT showed significant absorption of right lower lobe inflammation and good lung recruitment (Figure 2F). In both cases, adjunctive pulmonary rehabilitation was provided during convalescence, and both children recovered and were discharged.

## 3 Discussion

Prevotella pulmonary infection often presents with non-specific manifestations and can be easily overlooked. With advances in molecular diagnostics, Prevotella identification has shifted from traditional phenotypic methods to genome-based approaches; 16S rRNA gene sequencing and matrix-assisted laser desorption/ionization time-of-flight mass spectrometry (MALDI-TOF MS) are widely used for species-level identification (2). To contextualize our cases, we searched PubMed (title/abstract: “Prevotella” AND “lung disease”) and the China National Knowledge Infrastructure (CNKI) database (title/abstract: “pulmonary infection” AND “Prevotella”) for 2020–2025 and identified 7 and 2 relevant publications, respectively, including reports of severe pediatric cases.

Published reports of Prevotella-related pulmonary infection are largely case reports, some of which involve severe disease requiring intensive care or surgery. Reported risk factors include poor oral hygiene/periodontal disease, immunocompromise, and anatomic abnormalities. For example, Gong et al. (3) described Prevotella oralis sepsis with pulmonary aspergillosis in a patient with common variable immunodeficiency; Cobo et al. (4) reported two severe pleural infections (Prevotella oralis-associated septic shock in a man with mediastinitis and Prevotella gingivalis pleural infection in a woman with systemic lupus erythematosus). Other severe cases include refractory pneumonia due to Prevotella heparinolytica (5), lung abscess due to Prevotella salivae and Prevotella oralis (6), and pneumonia with parapneumonic effusion caused by Prevotella pleuritidis in an adolescent with diabetes (7). Severe infection can also occur in otherwise healthy individuals under specific circumstances, such as periodontitis (8) or poor oral hygiene (9). In our report, Case 1 occurred in the context of necrotizing pneumonia, whereas Case 2 was associated with a retained airway foreign body-both recognized high-risk settings for severe anaerobic infection.

Clinical manifestations of severe Prevotella pulmonary infection in children are non-specific. Systemic symptoms include persistent high fever and fatigue, while respiratory symptoms include severe cough, purulent sputum, and chest pain. Disease progression may lead to hemoptysis and dyspnea, and severe cases may be complicated by life-threatening conditions such as necrotizing pneumonia, lung abscess, empyema, and mediastinitis, requiring high clinical vigilance. To summarize clinical and therapeutic features, representative case reports and the two patients in this study are compared in Table 1.

**Table 1:** Summary of clinical characteristics of cases with Prevotella-associated pulmonary infection.

Reference	Sex	Age	Clinical presentation	Radiographic findings	Diagnostic method (Pathogen)	Antibiotic therapy (Sequence)	Outcome
Sun et al. (2024) (5)	M	58 years	Cough, white sputum for 20 days, fever for 3 days; history of periodontitis	Multiple patchy nodules in both lungs	mNGS (BAL fluid)	Initial: Moxifloxacin → Adjusted to: Piperacillin-Tazobactam+Ornidazole	Symptoms resolved, inflammation absorbed, good prognosis.
Gong et al. (2020) (3)	F	14 years	Cough and sputum for >3 months, recurrent infections, hypoglobulinemia	Bilateral patchy opacities with consolidation	Blood culture, sputum culture, bronchial aspirate culture	Initial: Cefamandole → Adjusted to Cefoperazone-Sulbactam → After positive blood culture: Metronidazole + Piperacillin-Tazobactam → + Voriconazole for Aspergillus fumigatus	Improved after anti-infective therapy and IVIG; required ongoing antifungal therapy.
Zeng et al. (2021) (8)	M	24 years	Cough, sputum for 1 week with chest pain; history of periodontitis	Multiple nodules and patchy opacities with cavity formation in both lungs	mNGS (BAL fluid)	Initial: Ceftriaxone → Adjusted to: Moxifloxacin monotherapy after mNGS	Marked resolution of lesions after therapy, good prognosis.
Martinez Castrejon et al. (2024) (9)	F	11 years	Persistent dry cough, right chest pain, fatigue, dyspnea, progressing to empyema	Right pleural effusion, right pneumothorax, sternocleidomastoid thickening on CT	Pus anaerobic culture	Initial: Vancomycin + Ceftriaxone → Adjusted to: Teicoplanin + Metronidazole (combined with VAC therapy)	Complete resolution of infection after 26 days of VAC therapy.
Li et al. (2024) (21)	F	56 years	Gingival pain, fever, progressing to neck abscess and severe pneumonia	Multiple patchy opacities in right lung with mediastinal lymphadenopathy	mNGS (Peripheral blood & pus)	Initial: Oral Metronidazole → IV Cefazolin + Metronidazole → Adjusted to: Meropenem + Pefloxacin after mNGS	Symptoms relieved, lung lesions absorbed after targeted therapy.
Galliguez et al. (2021) (7)	M	12 years	Chest pain, cough, dyspnea, fever (history of diabetes)	Right-sided pneumonia with large pleural effusion on x-ray/CT	Clinical mNGS (Explify platform)	Initial: Cefepime + Vancomycin → Ceftriaxone + Linezolid → Adjusted to: Meropenem → Oral Metronidazole	Clinical and radiographic improvement, discharged successfully.
Wu et al. (2024) (6)	M	67 years	Lung abscess symptoms (with lung cancer)	Right upper lobe lesion with local necrosis on CT	Third-generation mNGS (BAL fluid/aspirate)	Initial: Imipenem/Cilastatin + Vancomycin → Adjusted to: Clindamycin + Piperacillin/Tazobactam	Body temperature normalized, symptoms improved, CT showed resolution before discharge.
Cobo et al. (2022) [Case 1] (4)	M	60 years	Chest pain, malaise, vomiting, with mediastinitis	Extensive gas in neck and mediastinum with pleural effusion on CT	Pus MALDI-TOF MS & 16S rRNA sequencing	Piperacillin-Tazobactam + Linezolid	Rapidly progressed to septic shock, died on day 6.
Cobo et al. (2022) [Case 2] (4)	F	47 yrs	Somnolence, unable to speak or open eyes (SLE, myxedema coma)	Left pleural effusion on chest ultrasound	Pus MALDI-TOF MS & 16S rRNA sequencing	Meropenem (IV)	Discharged after 20 days of treatment.
Present Study [Case 1]	M	11 years	Recurrent cough, fever for >1 month with chest pain, hemoptysis; diminished breath sounds in right middle lobe	Consolidation in right middle lobe, pleural effusion, bronchial occlusion	mNGS (BAL fluid, Prevotella nanceiensis)	Initial: Levofloxacin → Adjusted after mNGS: Metronidazole + Meropenem	Improved after combined anti-infective and surgical treatment; follow-up CT showed inflammation resolved.
Present Study [Case 2]	M	13 years	Cough, fever, right lower chest pain; history of pen cap aspiration	Right lower lobe pneumonia, pleural effusion	mNGS (BAL fluid, Prevotella oralis)	Initial: Cefotaxime → Adjusted after mNGS: Metronidazole + Imipenem/Cilastatin	Improved after combined anti-infective therapy and thoracentesis; follow-up CT showed inflammation resolved.

As summarized in Table 1, periodontitis, poor oral hygiene, and foreign-body aspiration are common predisposing factors, creating conditions for invasion by this resident anaerobe. Second, the diagnostic process often involves ineffective empirical therapy with broad-spectrum antibiotics (e.g., fluoroquinolones or cephalosporins), highlighting the challenge of timely pathogen identification. Notably, when metagenomic next-generation sequencing (mNGS) was applied, rapid organism identification frequently prompted a switch to targeted anti-anaerobic therapy. Finally, successful regimens for severe or complicated infections typically include metronidazole, a carbapenem, or a beta-lactam/beta-lactamase inhibitor combination, underscoring the need for anaerobic coverage.

Anaerobic culture remains the reference standard for confirming anaerobic pathogens, but its turnaround time and limited sensitivity may delay diagnosis. In our Case 1, anaerobic culture became positive after 4 days. In contrast, metagenomic next-generation sequencing (mNGS) can identify pathogens without culture by sequencing total microbial DNA and typically returns results within 24–48 h. This rapid, culture-independent approach is particularly valuable in pediatric severe infections, where sample volumes are limited and disease progression can be rapid, enabling earlier etiologic diagnosis and timely adjustment of anti-infective therapy (10–13). Both patients in this report were diagnosed within 24 h by BAL mNGS. Studies in other severe infections have also shown higher detection rates with molecular sequencing than with conventional culture, including diabetic foot infection (14), brain abscess (15), and severe urinary tract infection (16, 17). Case 2 further illustrates mNGS value: Prevotella oralis was detected with high read counts and relative abundance in both BAL fluid and pleural effusion, supporting true infection rather than oral contamination or asymptomatic colonization. Nevertheless, mNGS may detect non-viable organisms or contaminants (18), so results should be interpreted in conjunction with clinical findings, imaging, and other laboratory tests. The subsequent positive anaerobic culture in Case 1 provided additional confirmation.

Both patients responded poorly to initial monotherapy with fluoroquinolones or cephalosporins, consistent with reported antimicrobial resistance in Prevotella spp. Although beta-lactams are commonly used, Prevotella may harbor resistance genes (e.g., tetQ, blaTEM, ermF) and can be resistant to beta-lactams and macrolides; metronidazole resistance has also been reported (19). For beta-lactamase-producing strains, beta-lactam/beta-lactamase inhibitor combinations (e.g., piperacillin-tazobactam) are recommended (20, 21), particularly in severe infections. Published case reports support escalation to anti-anaerobic combination therapy when empirical regimens fail (5, 22). In our two cases, with reference to the 2025 European Guidelines on Anaerobes, the antibiotics were adjusted to metronidazole plus a carbapenem (meropenem for Case 1 and imipenem-cilastatin for Case 2), with good clinical outcomes. Carbapenems should be reserved for critically ill patients or for failure of first-line regimens to limit unnecessary use and resistance selection. While metronidazole is effective against many anaerobes, resistance rates in oral Prevotella isolates have been reported and are often associated with nim genes (23). Therefore, combination therapy may be required for severe disease. When resistance testing is unavailable or results are inconclusive (24), empirical regimens with anaerobic coverage (metronidazole and/or a beta-lactam/beta-lactamase inhibitor combination) are reasonable, with carbapenems considered for severe infections (25, 26).

In both cases, adjunctive pulmonary rehabilitation was implemented and achieved favorable outcomes. Therefore, once oxygenation improves and the patient becomes afebrile with declining inflammatory markers, early pulmonary rehabilitation can be introduced to enhance airway clearance, facilitate lung re-expansion, and accelerate functional recovery—particularly in necrotizing pneumonia, pleural involvement, or after thoracic surgery. Key components include airway clearance techniques, breathing exercises, and graded early mobilization combined with shoulder and chest-wall range-of-motion training to prevent dysfunction.

## 4 Conclusion

Severe Prevotella pulmonary infection in children can be easily overlooked. Clinicians should pay attention to high-risk factors such as foreign-body aspiration, poor oral hygiene, and immunocompromise. Poor response to conventional beta-lactams or fluoroquinolones may be an important clue. For diagnosis, mNGS is rapid and sensitive and can overcome limitations of traditional culture, but results should ideally be integrated with anaerobic culture and clinical context. For treatment, monotherapy with fluoroquinolones or cephalosporins is often insufficient in severe cases; targeted regimens with anaerobic coverage are required. For severe or complicated infections, metronidazole combined with a beta-lactam/beta-lactamase inhibitor or a carbapenem should be considered based on etiologic evidence. Finally, multidisciplinary collaboration among pediatric pulmonologists, radiologists, and clinical microbiologists is essential for early recognition, accurate interpretation of mNGS results, rational antimicrobial selection, and improved outcomes.

## Data Availability

The original contributions presented in the study are included in the article/Supplementary Material, further inquiries can be directed to the corresponding author/s.
